# Discussion Required for Correct Interpretation

**DOI:** 10.1371/journal.pmed.0030369

**Published:** 2006-08-29

**Authors:** Radek Bukowski, Gary D. V Hankins, George R Saade, Steve Thornton

**Affiliations:** University of Texas Medical Branch, Galveston, Texas, United States of America; University of Warwick Coventry, United Kingdom

Thank you for the opportunity to comment on the editorial by Romero and colleagues [1], which raises a number of important and interesting questions. Such discussion is mandatory if results of scientific techniques such as gene array are to be correctly interpreted and used as the basis for future improvements in patient care.

It is interesting that re-analysis of our results does not demonstrate significant changes in gene expression and highlights the importance of the analysis technique in data interpretation. We agree with the editorial that there is a wealth of data that supports labour-associated changes in gene expression [2–5] and any implication that there are no significant labour-associated differences would ignore the results of numerous targeted analyses published by many independent researchers worldwide. Indeed the number and quality of such publications led us to make the basic assumption that there are labour-associated changes in gene expression. For this reason we calculated the *p*-value to identify those genes with the greatest difference in expression and smallest variability rather than to determine significance. This analytical design was strengthened by gene array analysis of each patient sample rather than analysis of pooled mRNA. We accept that there are a number of techniques available that can determine statistical significance whilst attempting to account for multiple analyses in gene array studies. However, those genes most likely to be important for the biological process of labour may or may not demonstrate a significant change in expression when corrections are made for multiple analyses. Furthermore, we have demonstrated that changes in the expression of individual genes are not independent, which complicates analysis. For example, the false discovery rate assumes a certain level of correlation among genes. However, the size of this correlation is not known and if underestimated can result in a high false negative rate. Thus, in keeping with other human uterine gene array data [6,7], we believe that there are likely to be important biological changes in labour-associated gene expression that may or may not be reflected by finding statistical significance of a test chosen for analysis. We agree that the preparation (activation) of the uterus for the onset of labour is likely to occur in the weeks preceding its onset and such changes cannot be expected to be identified by labour and non-labour comparisons of gene expression.

Our original analysis included patient data, which was removed during re-analysis by Romero et al. Data from one patient were omitted because the arrays demonstrated saturation. A second set of data was removed because the modal probe intensity was 8-fold higher than the others. We normalised our data by transforming the expression value into a percentile and giving this as a multiple of the standard deviation (*z*-score). Thus, in contrast to some other methods of transformation (e.g., logarithmic), ours is insensitive to the magnitude of expression since genes are given a rank relative to other genes. Data from a third patient were removed because a different Affymetrix chip was used. We considered that inclusion of data from this chip was appropriate since it is almost identical to the original (all apart from 25 of the 12,626 genes), and ranking genes by their *z*-scores removes chip-to-chip differences.

Our analysis identified genes that demonstrate a marked labour-associated difference in expression. We used this data to identify networks of genes that are co-regulated. We believe that the process of labour does not result from the independent expression of single genes but the effect of coordinated regulation of groups of genes that act in synchrony on a primed uterus to initiate labour.
